# Plasticity of Central Chemoreceptors: Effect of Bilateral Carotid Body Resection on Central CO_2_ Sensitivity

**DOI:** 10.1371/journal.pmed.0040239

**Published:** 2007-07-24

**Authors:** Albert Dahan, Diederik Nieuwenhuijs, Luc Teppema

**Affiliations:** Department of Anesthesiology, Leiden University Medical Center, Leiden, The Netherlands; University of California Los Angeles, United States of America

## Abstract

**Background:**

Human breathing is regulated by feedback and feed-forward control mechanisms, allowing a strict matching between metabolic needs and the uptake of oxygen in the lungs. The most important control mechanism, the metabolic ventilatory control system, is fine-tuned by two sets of chemoreceptors, the peripheral chemoreceptors in the carotid bodies (located in the bifurcation of the common carotid arteries) and the central CO_2_ chemoreceptors in the ventral medulla. Animal data indicate that resection of the carotid bodies results, apart from the loss of the peripheral chemoreceptors, in reduced activity of the central CO_2_ sensors. We assessed the acute and chronic effect of carotid body resection in three humans who underwent bilateral carotid body resection (bCBR) after developing carotid body tumors.

**Methods and Findings:**

The three patients (two men, one woman) were suffering from a hereditary form of carotid body tumors. They were studied prior to surgery and at regular intervals for 2–4 y following bCBR. We obtained inspired minute ventilation (V_i_) responses to hypoxia and CO_2_. The V_i_-CO_2_ responses were separated into a peripheral (fast) response and a central (slow) response with a two-compartment model of the ventilatory control system. Following surgery the ventilatory CO_2_ sensitivity of the peripheral chemoreceptors and the hypoxic responses were not different from zero or below 10% of preoperative values. The ventilatory CO_2_ sensitivity of the central chemoreceptors decreased by about 75% after surgery, with peak reduction occurring between 3 and 6 mo postoperatively. This was followed by a slow return to values close to preoperative values within 2 y. During this slow return, the V_i_-CO_2_ response shifted slowly to the right by about 8 mm Hg.

**Conclusions:**

The reduction in central V_i_-CO_2_ sensitivity after the loss of the carotid bodies suggests that the carotid bodies exert a tonic drive or tonic facilitation on the output of the central chemoreceptors that is lost upon their resection. The observed return of the central CO_2_ sensitivity is clear evidence for central plasticity within the ventilatory control system. Our data, although of limited sample size, indicate that the response mechanisms of the ventilatory control system are not static but depend on afferent input and exhibit a large degree of restoration or plasticity. In addition, the permanent absence of the breathing response to hypoxia after bCBR may aggravate the pathological consequences of sleep-disordered breathing.

## Abstract 2

Bilateral carotid body resection in three individuals led to reduced sensitivity of central chemoreceptors to CO_2_, followed by a gradual return, providing evidence of central plasticity within the ventilatory control system.

## Introduction

Human breathing is regulated by two control systems, behavioral control and metabolic control. They both make use of complex feed-forward and feedback control mechanisms allowing the strict matching between our metabolic and nonmetabolic needs and oxygen uptake in the lungs [1]. For example, behavioral control enables our breathing to adapt to various activities, such as speaking, singing, or eating. The metabolic ventilatory control system drives our breathing at rest and ensures optimal cellular homeostasis with respect to pH, partial pressure of carbon dioxide (Pco_2_), and partial pressure of oxygen (Po_2_). Metabolic control consists primarily of some yet-to-be-identified mechanism by which breathing is grossly matched to metabolic rate. It uses two sets of chemoreceptors that provide a fine-tuning function: the central chemoreceptors located in the ventral medulla and the peripheral chemoreceptors in the carotid body, a small (10 mm^3^, 15 mg) and highly vascularized organ situated in the bifurcation of the common carotid artery. The central chemoreceptors are sensitive to hypercapnia (high blood CO_2_ levels), and the peripheral chemoreceptors are sensitive to hypercapnia and hypoxia (low blood oxygen levels). Activation of the sensors by their respective stimuli results in brisk ventilatory responses aimed at the restoration of cellular homeostasis [1]. The carotid bodies are strategically situated in the carotid arteries and are sometimes referred to as the “watchdogs” of the brain [2].

Diseases of the carotid body are rare. Tumors of the carotid body are commonly associated with chronic tissue hypoxia due to living at altitude, cyanotic heart disease, and chronic pulmonary disease [3–6]. There are also congenital forms of carotid body tumors [7]. A hereditary form, which is relatively common in the Leiden region, is due to a missense mutation of the gene that encodes succinate dehydrogenase D (SDHD) [8]. SDHD is a small part of cytochrome b588 of the mitochondrial respiratory chain complex II and is an essential enzyme in the tricarboxylic acid cycle. These carotid body tumors are part of the hereditary paraganglioma type 1 (PGL1) syndrome. The gene responsible for this syndrome, *SDHD* (formerly *PGL1*), is located on the long arm of Chromosome 11 (11q22–23). In Dutch founder families a missense mutation (c.274G>T) was observed that caused a p.Asp92Tyr (replacement of aspartic acid by tyrosine at amino acid position 92) in the SDHD gene product [8]. Although carotid body tumors are slow-growing and benign, they can cause significant morbidity due to compression and destruction of large vessels and nerves in the head- and neck region [7]. Large carotid body tumors are therefore surgically removed to prevent such damage.

Removal of the carotid bodies, especially by bilateral resection, allows for a unique insight in the mechanisms and complexity of the ventilatory control system in otherwise healthy patients. Bilateral carotid body resection (bCBR) may, for example, shed light on a possible interaction between peripheral and central chemoreceptors or on the existence of neuroplasticity in response to the loss of carotid body function. We use the term plasticity defined as “a persistent change in the neural control system (morphology and/or function) based on prior experience” [9]. Data from goats, piglets, and rats show a significant loss of central chemoreceptor activity upon bCBR that is followed by a relatively rapid return to preoperative conditions due to central neural plasticity [10–13]. Whether the human brain is able to respond in a similar way to bCBR is not known but is plausible. There are various examples of change and restoration of function within the human central nervous system after brain and spinal cord injuries. For example, observed recovery of ventilation after severe (80%) destruction of the spinal cord at the C2 level has been ascribed to plasticity of the spared respiratory pathways [9,14]. Earlier human studies on carotid body resections were done in patients with severe asthma and dyspnea or with significant atherosclerosis and were single measurements often decades after the resections had been performed [15–19]. These studies yielded very limited information on the effect in the time domain of the loss of the carotid bodies on the ventilatory control system.

To determine the interactive effect of peripheral and central chemorecepors on breathing and the effect of bilateral resection of the peripheral chemoreceptors at the carotid bodies, three otherwise healthy patients who are carriers of the *SDHD* gene and had carotid body tumors were studied prior to surgery and at regular intervals 2–4 y after bilateral resection of their carotid body tumors. We obtained ventilatory responses to hypoxia and hypercapnia by applying 3-min hypoxic pulses and pseudo-random steps into and out of hypercapnia, respectively. Using a two-compartment mathematical model of the ventilatory control system, we partitioned the hypercapnic ventilatory responses into a fast dynamic component originating at the peripheral chemoreceptors and a slow dynamic component originating at the central chemoreceptors [14,20–25]. To our knowledge, this is the first study to assess the effect of bCBR in healthy patients over a prolonged period of time.

## Methods

### Patients

Three members of Dutch families with hereditary paragangliomas (two men, one women) who had developed bilateral carotid body tumors were included in the study. Their *SDHD* c.274G>T mutation carrier status was verified by direct sequencing. Since the most common PGL1 tumor locations are the carotid bodies and the adrenal medulla the patients were regularly checked for pheochromocytomas. Our three patients remained negative for pheochromocytomas during the study, did not smoke or use any medication, and had a body mass index below 25. Their carotid body tumors were removed one at a time with about 6 mo in between resections. The age of the patients at complete removal of the carotid bodies was 56 y (patient 1), 45 y (patient 2), and 36 y (patient 3). The Human Ethics Committee of Leiden University Medical Center approved the study protocol and each patient provided written informed consent, including for publication.

### Study Design and Apparatus

Prior to removal of the carotid bodies we tested the patients once. In one patient we performed respiratory studies also after unilateral resection (patient 3). After bilateral resection we tested the patients at regular intervals up to 2–4 y post-bCBR. During each test session we obtained the ventilatory response to carbon dioxide, the ventilatory response to hypoxia, and the resting ventilatory variables: resting P_ET_ co_2_ and resting inspired minute ventilation. In patient 1 we additionally obtained resting P_ET_ co_2_ values during the first 5 d postoperatively (i.e., post-bCBR). All studies were performed from 3 p.m. to 6 p.m.

The ventilatory responses to carbon dioxide and hypoxia were obtained using the “dynamic end-tidal forcing” technique [20,23]. This technique allows for the manipulation of end-tidal concentration of one gas while maintaining the end-tidal concentration of the other gas constant. During studies, participants were comfortably seated. They breathed through a face mask that was connected to a pneumotachograph/pressure transducer system (Hans Rudolph, http://www.rudolphkc.com/), from which we obtained inspired minute ventilation. The inspired gas mixture was set via a system of mass-flow controllers (Bronkhorst High-Tec, http://www.bronkhorst.com/), which received control signals from a computer. O_2_ and CO_2_ concentrations were continuously measured near the mouth with a Datex multicap gas monitor (Datex-Engstrom, http://www.instrumentarium.com/worldwide.html); arterial oxygen–hemoglobin concentrations (Spo_2_) were measured with a pulse oximeter using a finger probe (Masimo, http://www.masimo.com/). Prior to each session the pneumotachograph and gas monitor were calibrated using a motor-driven piston pump and gas-mixing system (Wösthoff, http://woesthoff.com/), respectively.

#### The dynamic ventilatory response to carbon dioxide.

The ventilatory responses to carbon dioxide were separated into a peripheral and central component (see Data Analysis section). In order to allow separation we optimized the end-tidal partial pressure of carbon dioxide (P_ET_ co_2_) input function. We applied pseudo-random increases and decreases in P_ET_ co_2_) and measured the ventilatory response on a breath-to-breath basis. We used a multifrequency binary sequence (MFBS) in P_ET_ co_2_ involving 26 steps into and 26 steps out of hypercapnia (total duration 47 min, see Figure 1) [21,22,24]. Low and high CO_2_ levels were +3 and +10.5 mm Hg above the patients' resting values. The MFBS was developed to spread its power over the frequency range of interest to optimize identification of both the peripheral and central chemoreceptor ventilatory responses. The MFBS has been used in both physiological and pharmacological studies [21,24]. In the current study we doubled length of the MFBS over that used in previous studies. It is our experience that the precision of model parameters, but especially that related to central chemoreceptors, increases when using the double sequence [24].

**Figure 1:**
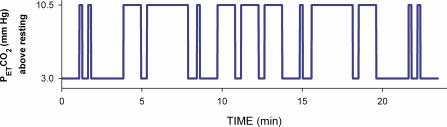
The Multifrequency Binary Sequence in P_ET_ co_2_ In this study we applied two of the shown (approximately 23.5 min) sequences in succession.

#### The acute hypoxic ventilatory response.

We applied three 3-min hypoxic episodes in sequence, with a 3-min normoxic interval between hypoxic episodes. Each episode consisted of a step from normoxia (Spo_2_ 99%) into hypoxia (Spo_2_ 80%), which was obtained by manipulation of the end-tidal oxygen concentration (P_ET_ o_2_). On average the P_ET_ o_2_ needed to obtain a Spo_2_ of 80% was 43 mm Hg. During these studies the P_ET_ co_2_ was kept constant at +2 mm Hg above resting.

### Data Analysis

#### The dynamic ventilatory response to carbon dioxide.

The data were analyzed by fitting the ventilatory response to a linear two-compartment model of the ventilatory control system, as described previously. The steady-state relation of inspired ventilation (V_i_) to P_ET_ co_2_ at constant P_ET_ o_2_ in humans is linear and described by [1,20]:

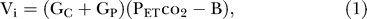
where G_C_ and G_P_ are the ventilatory CO_2_ sensitivities of the central and peripheral chemoreceptors, respectively, and B is the extrapolated P_ET_ co_2_ of the steady-state ventilatory response to CO_2_ at which ventilation is zero (apneic threshold; see Figure 2). Separation of the response into a peripheral and central component is possible since the outputs of the peripheral and central chemoreceptors display large differences in the time domain. The ventilatory output of peripheral chemoreceptors in response to stimulation with CO_2_ increases rapidly (time constant of 3–10 s), while the response of the central chemoreceptors is much slower (time constant of about 2 min). Furthermore, when applying a step in P_ET_ co_2_ at the mouth, the peripheral chemoreceptors will respond with a delay of about 6–8 s, while the central chemoreceptors have a delay of about 12–24 s [1,20,25]. See also Figure 3 for an explanation. In our model we included a linear trend term and a parallel noise component to model the noise corrupting the ventilation data [1,20]. Estimation of the parameters was performed with a one-step prediction error method [26]. The two-compartment model has been validated extensively in animals and humans [21,27,28]. In our current report we focus on the two more important parameters of our model: the ventilatory CO_2_ sensitivities of the peripheral and central chemoreceptors (G_P_ and G_C_).

**Figure 2:**
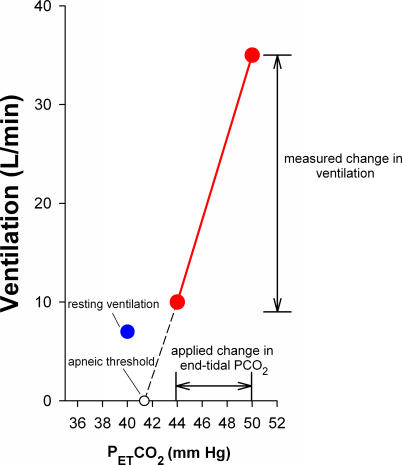
An Example of the Steady-State Ventilatory Response to an Increase in P_ET_ co_2_ from 44 to 50 mm Hg The response is linear (continuous red line) over the given trajectory; the extrapolated P_ET_ co_2_ (red circles) at which ventilation is zero is given (in this example it is 41.3 mm Hg, open symbol). Resting ventilation and resting P_ET_ co_2_ are also shown (blue circle).

**Figure 3:**
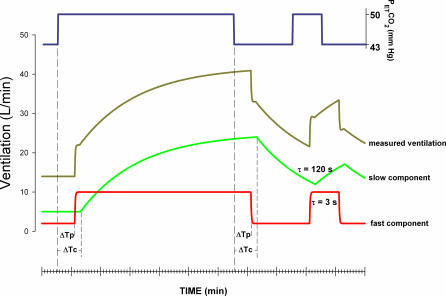
Separation of the Dynamic Ventilatory Response to P_ET_ co_2_ P_ET_ co_2_ is depicted in blue. Shown is separation of measured ventilation (dark yellow) into a fast component with a short delay (ΔT_P_) originating at the peripheral chemoreceptors (red, time constant of response 3 s), and a slow component with a long delay (ΔT_C_) originating at the central chemoreceptors (green, time constant of response 120 s).

After the analysis with the two-compartment model we also analyzed the data with a single compartment model by fixing G_P_ to zero. We compared the two models (one compartment versus two compartments) using the Akaike Information Criterion (AIC) [29]. The model with the lowest AIC was considered best. This enabled us to assess whether the fast peripheral component was significantly different from zero. Goodness of fit was assessed by inspection of the data fit by eye, the autocorrelation function of the residuals, the cross-correlation function between stimulus (in our study P_ET_ co_2_), and residuals and the AIC [26,29].

#### The acute hypoxic ventilatory response.

We averaged the ventilation and Spo_2_ data of the final ten breaths of normoxia (prior to hypoxia) and hypoxia. The average change in ventilation was divided by the average change in Spo_2_ to get the hypoxic sensitivity (units, liters per minute per % desaturation [l·min^−1^·%^−1^]).

## Results

In all three patients, control ventilatory responses to carbon dioxide were in the mid-range of “normal” study populations (normal value approximately 1.5 l·min^−1^·mm Hg^−1^ [20,22]). The values of the contribution of the peripheral chemoreceptors to total ventilation were at the low end of what is normally observed in healthy volunteers. In our three participants, we observed that the contribution of the peripheral response to the total response to CO_2_ was on average 22% (range 16%–31%). In healthy volunteers this value ranges from 20% to 40% [20,28]. Similarly, the preoperative values of the ventilatory responses to the 3-min hypoxic pulses were at low end of “normal” with a mean response of 0.3 l·min^−1^·%^−1^. In normal volunteers the hypoxic ventilatory response ranges from 0.2–2.0 l·min^−1^·%^−1^ [22,30].

Removal of just one carotid body (patient 3) reduced the peripheral contribution to the ventilatory response to CO_2_ by 50% without reducing the ventilatory response to hypoxia (Table 1), indicating that compensation by the contralateral carotid body occurred with respect to the response to hypoxia but not to hypercapnia. The activity of the central chemoreceptors was reduced by 40% after unilateral carotid body resection (Table 1).

**Table 1:**
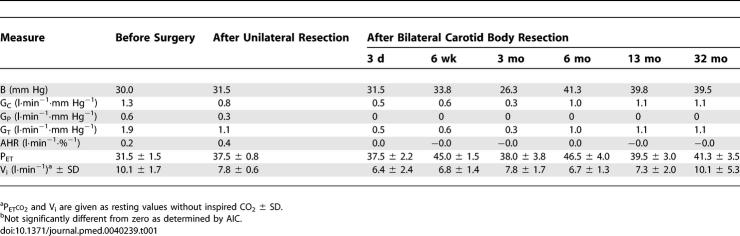
Resting Variables and Parameters Obtained from the CO_2_ and Hypoxic Challenges of Patient 3 over Time

Bilateral resection of the carotid bodies resulted in an increase in resting P_ET_ co_2_ values of 6–8 mm Hg. This effect occurred within the first days after surgery in patients 1 and 3 but developed more slowly in patient 2 (Figure 4; Tables 1–3). These changes in P_ET_ co_2_ were accompanied by a reduction in resting ventilation in two of the patients (1 and 3; Tables 1 and 3). After bCBR the dynamic ventilatory responses to CO_2_ did not contain a fast peripheral component or yielded a component that was less than 10% of the preoperative value. Reanalysis of the ventilatory data that contained a fast component with a one-compartment model (G_P_ fixed to zero) indicated that in nine of these 13 responses the fast component was not different from zero (Tables 1–3). The relative small but significant values of G_P_ observed in the remaining four responses may arise from the combination of imperfections in the model of the dynamic central component and noise. After bCBR none of the patients displayed an increase in ventilation during exposure to 3 min of hypoxia, neither initially nor after a period of 2–4 y.

**Figure 4:**
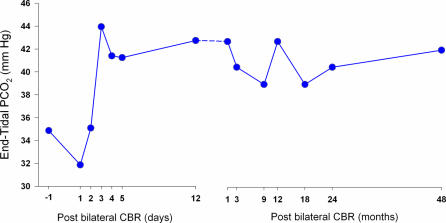
Resting P_ET_ co_2_ Values Observed on Days 1–5 after Bilateral Resection of the Carotid Bodies and Subsequent Days of Testing in Patient 1 The lower P_ET_ co_2_ values on postoperative day 1 may reflect anesthesia- or analgesia-induced lowering of metabolism. The subsequent rise in resting P_ET_ co_2_ is related to the loss of the peripheral chemoreceptors and the effect of this loss on the central chemoreceptors.

**Table 2:**
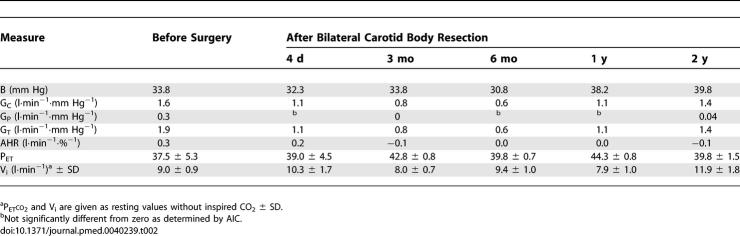
Resting Variables and Parameters Obtained from the CO_2_ and Hypoxic Challenges of Patient 2 over Time

**Table 3:**
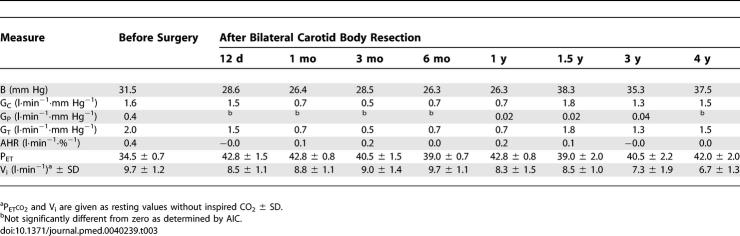
Resting Variables and Parameters Obtained from the CO_2_ and Hypoxic Challenges of Patient 1 over Time

Removal of the two carotid bodies had a pronounced depressant effect on the activity of the central chemoreceptors, with peak reduction in the magnitude of the response to CO_2_ occurring within 6 mo after bCBR (Figures 5 and 6). At peak depression the central responses had a magnitude of just 30% of preoperative values. Thereafter, the central component slowly increased to values in the range of values observed preoperatively (88% of preoperative value in patient 1 after 2 y, 85% in patient 3 after 13 mo, and over 100% after 18 mo in patient 2 [Figure 6; Tables 1–3]). We previously observed in healthy volunteers that due to normal biological variability the measured values of G_C_ range between 80% and 120% over a 4 y time period (data from [28,31,32]).

**Figure 5:**
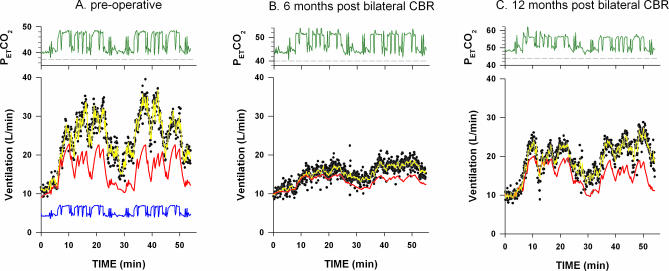
The Dynamic Ventilatory Response to CO_2_ in Patient 2 Response was monitored before resection of the carotid bodies (A), 6 mo after bilateral carotid body resection (B), and 1 y after bCBR (C). The applied multifrequency binary sequence is shown on the top of each graph (green; units mm Hg; the dashed lines represent resting P_ET_ co_2_ values). The breath-to-breath ventilation is depicted by the grey dots. The ventilatory response is separated into a slow component originating at the central chemoreceptors (red) and a fast component originating at the peripheral chemoreceptors (blue). The sum of these two components plus a trend term (not shown) and the (parallel) noise corrupting the data, is the yellow line through the measured data points. The analysis shows that 6 mo after resection of the two carotid bodies the peripheral component was not present and the central component initially reduced to about 40% of preoperative values. Six months later, the central component had increased to about 70% of preoperative values. Parameter values are given in Table 2.

**Figure 6:**
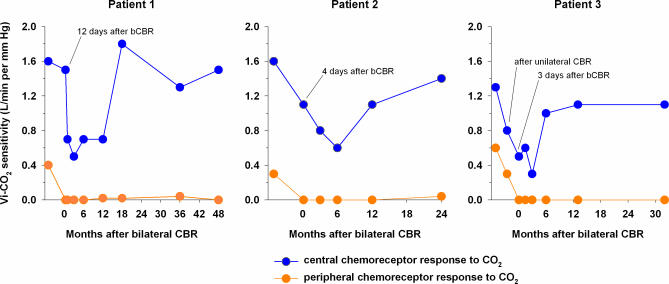
Central and Peripheral Chemoreceptor Activity in Response to Stimulation with CO_2_ in the Three Patients during the Whole Testing Period In all three patients bCBR caused the loss of the peripheral component or reduction to a value of less than 10% of preoperative values combined with an initial sharp reduction in the magnitude of the central component to about 30% of preoperative values, which was followed by a slow increase to values 85%–95% of the preoperative values. In patient 3 we also obtained data after unilateral carotid body resection.

The quality of the data fits was good, with white residual noise and small cross-correlations. Examples of the data fits are given in Figure 5. It shows, for patient 2, that prior to surgery a fast peripheral and a slow central component was identified. At 6 and 12 mo after surgery, the responses are smaller and only the central component was identified in the statistical model.

## Discussion

To our knowledge for the first time, the effects of bCBR on respiration were followed over time in healthy humans. Loss of the carotid bodies, which contain the peripheral chemoreceptors, results in loss of the ability to respond to hypoxia. Indeed, none of our three patients displayed a significant response to lowered inspired oxygen concentrations after bCBR (Tables 1–3). Apart from their involvement in the ventilatory response to hypoxia, the peripheral chemoreceptors of the carotid bodies play an appreciable role in the ventilatory response to carbon dioxide and as such determine an important part of the level of Pco_2_ in blood and body tissues. In humans with intact carotid bodies, the peripheral chemoreceptors contribute 20%–40% to the total ventilatory response to CO_2_ with the central chemoreceptors providing the remaining 60%–80% of ventilatory CO_2_ drive [1,20,22]. In agreement with the loss of the hypoxic ventilatory response, all of our three patients lost the fast component of the ventilatory response to CO_2_ and increased their P_ET_ co_2_ (which is a good reflection of arterial Pco_2_ in persons with normal lung and heart function) by 6–8 mm Hg. This pinpoints the origin of the acute isocapnic hypoxic ventilatory response (AHR) and the fast component in the CO_2_ response to the carotid bodies. It also adds proof to the validity of the two-compartment model we used in the data analysis.

The absence of recovery of the peripheral responses to hypoxia over time in the adult humans in this study indicates that other tissues, such as the aortic bodies in the wall of the aortic arch or other paraganglia (e.g., the vagal body in the neck), did not take over any function of the carotid bodies. Animal data suggest some return of the peripheral responses in neonatal (but not adult) rats and piglets from other sites after bCBR [11,12].

A remarkable observation was that the loss of the carotid bodies coincided with a sharp reduction in the ventilatory response to CO_2_ of the central chemoreceptors that peaked about 6 mo after surgery (Figure 6). Until now, it was assumed that in humans the peripheral and central chemoreceptor systems act independently and that their responses are additive [1,20,22]. For example, increasing the output of the peripheral chemoreceptor response to CO_2_ by inspiration of low oxygen concentrations or decreasing the output by subanesthetic concentrations of volatile anesthetics does not change the response of the central chemoreceptors to CO_2_ stimulation [17,20,28,31,32], suggesting the absence of a dynamic interaction between the two sensors. However, our present data suggest that the carotid bodies exert a tonic drive or tonic facilitation on the central chemoreflex loop that is lost upon their resection. Our results are in agreement with observations in goats, rats, and dogs, which display reduced ventilatory drive (reduced central CO_2_ sensitivity and increased arterial Pco_2_) after bCBR [10,12,33]. Various regions in the brain involved in breathing control are affected by bCBR in these animals. In goats bCBR is accompanied by a reduced ventilatory response to focal acidosis in the medullary raphe (a brain area containing chemosensitive serotonergic neurons) [34]. In adult rats, normocapnic hypoxia did increase discharge rates in CO_2_ sensitive neurons within the retrotrapezoid nucleus, an effect that was absent after peripheral chemoreceptor denervation [35]. These animal studies corroborate our findings and provide further evidence for a tonic facilatory input from the carotid bodies into various respiratory centers in the brain, including some that may contain the central chemoreceptors. In the rat, regions within the nucleus tractus solitarius receiving input from the carotid bodies have glutamatergic connections with the retrotrapezoid nucleus, a major chemosensitive area in the ventral medulla that projects to neighboring areas containing respiratory neurons [36]. If these connections also exist in humans, bCBR could have resulted in a reduced tonic facilatory output from the nucleus tractus solitarius to the retrotrapezoid nucleus.

The most important finding in our study was the slow recovery or return of the output of the central chemoreceptors after bCBR. This result is a clear evidence for neuroplasticity within the ventilatory control system. These new observations indicate that the human ventilatory control system is not static but highly plastic with almost full restoration of central CO_2_ sensitivity. The lost peripheral CO_2_ sensitivity was not restored in this process. The increased central CO_2_ sensitivity was unable to restore the increased P_ET_ co_2_ (from the loss of the carotid bodies) or cause a consistent increase in resting ventilation in 2 of our 3 patients to preoperative values (Figure 4; Tables 1 and 3). As a consequence, steady-state ventilation operates at a new setpoint with the apneic threshold about 8 mm Hg greater than before bCBR. In animals, data on the return of CO_2_ sensitivity after bCBR are equivocal, with full return observed in goats but not in dogs [10,13,33]. This variation may suggest a difference in the occurrence of central plasticity among species, although it is possible that in dogs the process is slower than in goats and possibly even slower than in humans, and thus was missed in the 21 d time frame of the dog study. The recovery observed in goats was relatively fast (within 15 d) [10], which suggests a change or shift in neurotransmitter turnover as the most plausible cause for the return of central CO_2_ sensitivity. In humans, the very slow return excludes a simple mechanism. Possible mechanisms include up-regulation of synaptic activity from growth (sprouting) or activation of silent synapses, synaptic remodeling, rerouting of afferent respiratory information, reorganization of respiratory centers, and/or recruitment of different neuronal centers [9]. It may well be that after bCBR a slow process is initiated that activates dormant CO_2_-sensitive neurons and incorporates them into the central chemoreceptor network. For example, CO_2_-sensitive neurons that are active only during specific arousal states (such as in the midline raphe nuclei during sleep, but also in the locus coeruleus, the nucleus tractus solitarius or the cerebellum in other conditions) may be recruited in a state-independent manner after bCBR (compare [37]).

It is important to discuss whether some of our observations were related to the mutation in the gene encoding SDHD. The ventilatory CO_2_ responses (including the estimated peripheral and central components) and AHR were of similar magnitude or at the low end of those measured in “normal” volunteers of the same age and gender [20,22,30]. SDHD is part of complex II of the mitochondrial electron transport chain [8]. The mitochondrial respiratory chain enzymes have been implicated in the mechanism of oxygen sensing at the carotid bodies, however, with only indirect evidence for involvement of SDHD [30,38,39]. In vitro studies on carotid bodies from heterozygous SDHD knockout mice (*SDHD* ^+/−^) indicate normal responsiveness to hypoxia despite abnormal enhancement of resting carotid body activity [40]. How this translates to the in vivo situation and to humans remains unknown. The observation of normal to near-normal peripheral CO_2_ responses before surgery suggests that the mechanism of CO_2_ sensing in the carotid body cells is unaffected in our patients or at best only mildly affected, and that the neuromechanical link between the carotid bodies and the ventilatory output is intact. Furthermore, we recently observed normal ventilatory responses to CO_2_ in *SDHD* ^+/−^ mice compared to their wild-type littermates (unpublished data). We therefore argue that the observed changes in central CO_2_ sensitivity in our three patients (that is, the reduction upon bCBR and the following recovery) were due to the loss of the carotid bodies and were unrelated to the c.274G>T mutation in the *SDHD* gene.

The changes we observed in our patients may have important implications for breathing during sleep. During sleep, particularly during non-REM sleep, breathing is critically dependent on metabolic control of breathing (behavioral control is absent) and thus on the relationship between ventilation and arterial Pco_2_. In our patients this relationship (Figure 2), as well as resting Pco_2_, was shifted to the right. High resting P_ET_ co_2_ values (which increase further during sleep) may cause irregular breathing, periodic breathing, and central sleep apnea during non-REM sleep when resting P_ET_ co_2_ values cross the apneic threshold due to subtle changes in ventilation [41]. In all three patients the difference between the apneic threshold and resting P_ET_ co_2_ was less than 1 mm Hg on various postoperative occasions (Tables 1–3), making them prone to the development of unstable breathing during non-REM sleep. Further study of breathing patterns in these patients may give us further insights in the mechanism of central sleep apnea.

Carotid body resection is not uncommon. We presented three cases in which the carotid bodies were resected because of bilateral tumor formation due to a familial genetic defect. Other more common causes of (bilateral) carotid body tumors are sporadic genetic defects, cyanotic heart disease, and pulmonary disease [3–5]. At high altitudes the frequency of carotid body tumors is ten times that of those living at sea level [6]. And even more important, carotid body resection is performed in patients who do not have carotid body tumors but do have asthma or COPD with severe dyspnea or after endarteriectomy in the carotid artery in severe artherosclerotic stenosis [15–19]. Our data indicate that in all of these cases the ventilatory response to hypoxia is abolished permanently, and the central CO_2_ sensitivity is greatly reduced for at least 6–12 mo. Especially the ventilatory response to hypoxia is considered a crucial chemoreflex in various conditions, such as at altitude and during apneic episodes in sleep from central or obstructive sleep apnea. In apneic episodes during sleep, hypoxia-induced carotid body activation causes arousal within the reticular formation, which is crucial in determining the moment at which (and the extent to which) breathing resumes. Absence of this mechanism in bCBR patients may lead to higher morbidity and eventually higher mortality due to the abnormally long apneic episodes. This is especially important because patients after bCBR are more prone to develop central sleep apnea (see above).

In conclusion, our data, although of limited sample size, indicate that the response mechanisms of the ventilatory control system are not static but depend on afferent input and exhibit a large degree of restoration or neuroplasticity. There are no reasons to assume that neural plasticity is not a general property of the adult central nervous system, especially after neurological trauma/injury. Our healthy patient group may serve as a unique model for the study of plasticity in a healthy brain.

## Supporting Information

### Accession Numbers

The GenBank (http://www.ncbi.nlm.nih.gov/) GeneID number of the *SDHD (PGL1)* gene is 6392.

**Editors' Summary**

**Background.**

Several complex mechanisms control human breathing (the rhythmic inspiration and expiration of air into and out of the lungs), the most important of which is the metabolic ventilatory (breathing) control system. Regular breathing ensures that enough oxygen (O_2_) is absorbed into the blood by the lungs to fuel the body's metabolism (the essential chemical processes of life). It also ensures that sufficient carbon dioxide (CO_2_), a by-product of metabolism, is removed from the blood to prevent its acidity increasing to a dangerous level. The central control area for breathing is in the medulla of the brainstem, which connects the brain to the spinal cord. Nerve cells in the medulla integrate information coming from the body and then send messages back that set the breathing rate. For example, during exercise, when blood CO_2_ levels are high (hypercapnia) and blood O_2_ levels are low (hypoxia), these neurons increase the breathing rate to correct these levels. Blood O_2_ and CO_2_ levels are detected by groups of cells called chemoreceptors, which transform this information into electrical messages that pass to the medulla. Central chemoreceptors in the brainstem respond to hypercapnia; peripheral chemoreceptors in the carotid bodies (two small organs in the arteries supplying the head and the neck with oxygenated blood) are sensitive to both hypercapnia and hypoxia. Especially the peripheral chemoreceptors play a crucial role in normalizing blood gas values during exercise.

**Why Was This Study Done?**

The human metabolic ventilatory control system is poorly understood. An improved understanding might help physicians treat respiratory conditions, such as sleep apnea (in which patients briefly and repetitively stop breathing while they are asleep). It might also help them care better for patients whose carotid bodies have been removed because of carotid body tumors. These benign tumors, which can be caused by a rare condition called hereditary paraganglioma, have to be removed because they compress and destroy vessels and nerves in the head and neck. In this study, the researchers investigated the effects on the metabolic ventilatory control system of removal of the carotid bodies in three otherwise healthy patients with hereditary paraganglioma.

**What Did the Researchers Do and Find?**

The researchers measured the patients' breathing responses to hypoxia and hypercapnia before removal of their carotid bodies and at regular intervals after surgery for up to 4 years. In one patient, measurements were also made after removal of one carotid body. They then used a mathematical model of the metabolic ventilatory control system to separate the ventilatory responses to CO_2_ into a peripheral and a central response. The ventilatory responses of all the patients were normal before surgery. Removal of one carotid body reduced the peripheral ventilatory response to CO_2_ by 50% but did not affect the response to hypoxia. After removal of both carotid bodies, the peripheral ventilatory response to both hypercapnia and hypoxia was rapidly, completely, and permanently lost. In contrast, the central ventilatory response gradually reduced until, by 3–6 months after surgery, it was at a quarter of its presurgery level. It then gradually returned to its preoperative level.

**What Do These Findings Mean?**

Although based on only three patients, these findings suggest that the peripheral chemoreceptors in the carotid bodies normally influence the output of the central chemoreceptors. Thus, ventilatory responses to CO_2_ are reduced directly by the removal of the carotid bodies and indirectly by the loss of CO_2_ sensitivity in the central chemoreceptors. In addition, the eventual recovery of the central sensitivity to CO_2_ indicates plasticity (adaptation) in the central chemoreceptors, an important result that suggests that the CO_2_ response mechanisms of the human ventilatory control system are not static. However, the finding that the three patients became permanently insensitive to hypoxia indicates that the ventilatory control system cannot adapt to reinstate sensitivity to low blood O_2_. Obstructive sleep apnea (which is associated with being overweight) in these patients might, therefore, lead to longer periods of breathing cessation than in patients with intact carotid bodies. Because longer apnea increases the risk of heart injury or stroke, physicians need to react quickly if sleep apnea develops in these patients.

The academic editor, Ronald Harper, who advised on the research article, commented as follows.

The findings are of interest to clinicians managing patients with heart failure, who must contend with a loss of cerebral vasoreactivity, the ability of brain vessels to dilate or constrict in response to CO_2_. It is unclear why that loss occurs, but decreased sensitivity of peripheral chemoreceptors, resulting from atherosclerosis of the carotid arteries blunting responses, may contribute. The impaired vasoreactivity in heart failure leads to an inability to adequately perfuse the brain in essential sites, possibly underlying the recently described injury in areas that themselves regulate breathing responses to CO_2_, blood pressure, and cognition, all serious problems for heart failure patients. The findings here suggest that recovery from peripheral chemoreceptor damage occurs, but requires such a long time period that significant brain injury may result. Dyspnea, the overwhelming perception of breathlessness with activity often experienced by patients with pulmonary disease or heart failure, is sensitive to rapidly changing CO_2_ levels detected by peripheral chemoreceptors. Insights into plasticity and interactions with central chemosensitivity following removal of peripheral CO_2_ sensors are especially valuable for understanding the dyspneic condition. In addition, a large number of infants are subjected to unilateral carotid body resection as an unintended consequence of extracorporeal membrane oxygenation (ECMO), a procedure used to oxygenate the body in the absence of adequately functioning lungs. It is unclear whether comparable patterns of chemosensitivity responses parallel the adult findings described here, but a description of such patterns should be the object of research efforts.

**Additional Information.**

Please access these Web sites via the online version of this summary at http://dx.doi.org/10.1371/journal.pmed.0040239.

• Wikipedia has pages on chemoreceptors carotid bodies, and paragangliomas (note: Wikipedia is a free online encyclopedia that anyone can edit; available in several languages)

• The MedlinePlus encyclopedia contains pages on sleep apnea and on central sleep apnea  (in English and Spanish)

• A brief article on the control of breathing is available from the journal *Physiology*

• Information on the effect of anesthesia, pain, and opioids (morphine and other potent pain killers) on breathing may be found at the Web site of the Control of Breathing Research Group, which includes some of the authors on this article as well as other researchers

## Abbreviations

AHR: acute isocapnic hypoxic ventilatory response
AIC: Akaike Information Criterion
bCBR: bilateral carotid body resection
MFBS: multifrequency binary sequence
Pco_2_: partial pressure of carbon dioxide
P_ET_co_2_: end-tidal partial pressure of carbon dioxide
P_ET_o_2_: end-tidal partial pressure of oxygen
Po_2_: partial pressure of oxygen
SDHD: succinate dehydrogenase subunit D
Spo2: arterial oxygen–hemoglobin saturation derived from pulse oximetry
V_i_: inspired minute ventilation
